# Productivity and apprenticeship employment intensity in Scotland: A longitudinal study at the enterprise level

**DOI:** 10.23889/ijpds.v8i2.2350

**Published:** 2023-09-14

**Authors:** Alma Sobrevilla, Zoe Mackay, Martyna Walczak, Malcolm Greig

**Affiliations:** 1 Skills Development Scotland, Glasgow, United Kingdom

## Objectives

We investigate the relationship between apprenticeship employment and productivity. Creating a new linked dataset allowed us to explore this research question for Scotland for the first time. Skills Development Scotland (SDS) holds data on employers of Modern Apprentices (MAs), but does not collect industry, size or economic performance measures such as Gross Value Added (GVA). Therefore, it was necessary to match our employer records to the Inter Departmental Business Register (IDBR) using Enterprise Reference Number (ERN).

## Methods

The Office for National Statistics (ONS) identified multiple matches for many Company IDs, so a cleaning process was required to identify a single match for each record. This involved matching records based primarily on company name and address.

We carried out Random Effects, Fixed Effects and System Generalised Method of Moments (GMM) regressions to analyse the relationship between productivity (real GVA per worker) and apprenticeship employment intensity (number of in-training apprentices as a proportion of total employment).

## Results

When we summarise the final dataset by enterprise, 42,486 company IDs were matched to 19,180 unique enterprises. We were able to link our SDS MA employer dataset to the following ONS datasets: Annual Business Survey, Business Register and Employment Survey, Business Structure Dataset, Business Enterprise Research and Development, Labour Force Survey, Employer Skills Survey and data on Producer Price Index.

Using this matched dataset we found a significant positive relationship between productivity and apprenticeship employment, which is robust to the inclusion of enterprise-level fixed effects (factors that are specific to each enterprise that could affect productivity but that do not change over time) and the use of a System GMM framework.

## Conclusion

Our results suggest that enterprises with a high proportion of apprentices are more productive, even after controlling for enterprise and industry-level characteristics. In order to study this relationship, it was crucial to construct a matched dataset containing information from different sources (SDS and ONS datasets).

